# 5-(3′,4′-Dihydroxyphenyl-γ-valerolactone), a Major Microbial Metabolite of Proanthocyanidin, Attenuates THP-1 Monocyte-Endothelial Adhesion

**DOI:** 10.3390/ijms18071363

**Published:** 2017-06-26

**Authors:** Charles C. Lee, Jong Hun Kim, Ji Seung Kim, Yun Sil Oh, Seung Min Han, Jung Han Yoon Park, Ki Won Lee, Chang Yong Lee

**Affiliations:** 1 Department of Food Science, Cornell University, Ithaca, NY 14853, USA; ccl93@cornell.edu; 2 Research Institute of Agriculture and Life Sciences, Seoul National University, Seoul 08826, Korea; j5nghun.kim@gmail.com (J.H.K.); junedong9@snu.ac.kr (J.H.Y.P.); 3 Major in Biomodulation, Department of Agricultural Biotechnology, Seoul National University, Seoul 08826, Korea; jiseung720@gmail.com (J.S.K.); osymi286@naver.com (Y.S.O.); heredar.doux@gmail.com (S.M.H.)

**Keywords:** 5-(3′,4′-dihydroxyphenyl-γ-valerolactone), proanthocyanidin microbial metabolite, atherosclerosis prevention, monocyte-endothelial adhesion

## Abstract

Several metabolomics of polymeric flavan-3-ols have reported that proanthocyanidins are extensively metabolized by gut microbiota. 5-(3′,4′-dihydroxyphenyl)-γ-valerolactone (DHPV) has been reported to be the major microbial metabolite of proanthocyanidins. We demonstrated that DHPV has stronger prevention effect on tumor necrosis factor (TNF)-α-stimulated adhesion of THP-1 human monocytic cells to human umbilical vein endothelial cells compared to its potential precursors such as procyanidin A1, A2, B1 and B2, (+)catechin, (−)epicatechin and its microbial metabolites such as 3-(3,4-dihydroxyphenyl)propionic acid and 2-(3,4-dihydroxyphenyl)acetic acid. Mechanism study showed that DHPV prevents THP-1 monocyte-endothelial cell adhesion by downregulating TNF-α-stimulated expressions of the two biomarkers of atherosclerosis such as vascular cell adhesion molecule-1 and monocyte chemotactic protein-1, activation of nuclear factor kappa B transcription and phosphorylation of I kappa-B kinase and IκBα. We suggested that DHPV has higher potentiality in prevention of atherosclerosis among the proanthocyanidin metabolites.

## 1. Introduction

Cardiovascular diseases (CVDs) are one of the major causes of death globally, which are mostly associated with the development of atherosclerosis. Atherosclerosis is a chronic inflammatory disease which involves the recruitment and accumulation of macrophages for the development of plaques [1]. It is known that these macrophages differentiate from monocytes recruited from circulating blood, and monocyte adhesion to endothelial cells is an important initial step of the development of atherosclerosis [1].

Retrospective and interventional studies have shown that the high intake of flavonoids prevent CVDs [2,3,4]. A meta-analysis of randomized controlled trials conducted by Hooper et al. (2008) also showed that the consumption of flavonoid-rich foods such as chocolate, cocoa and black tea strongly correlates with a reduced risk of CVDs [5]. These specific foods are known to be a source rich in flavan-3-ols comprised of monomeric and polymeric compounds [5]. Despite promising findings in clinical studies, it remains unclear which bioavailable compounds derived from flavan-3-ols exert the most potent preventive effects against CVDs.

**Figure 1:**
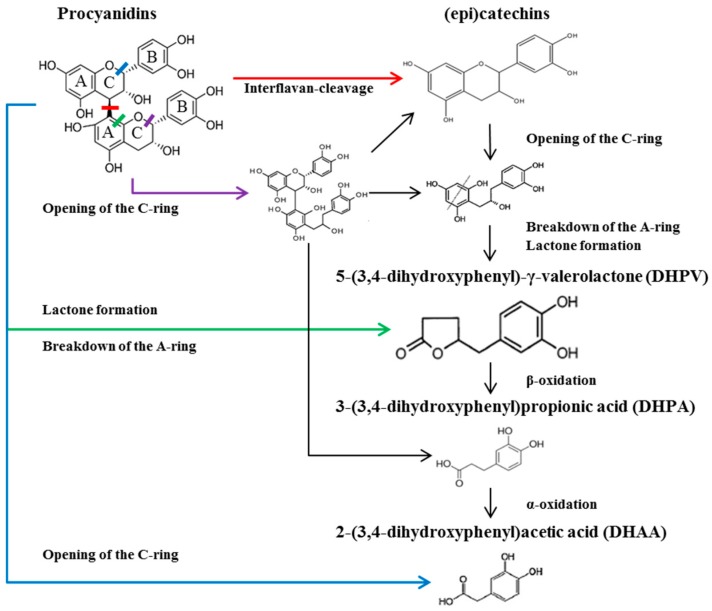
Microbial catabolism of procyanidins.

Flavan-3-ols are subjected to several metabolic pathways within the human body [6]. The bioavailability of flavan-3-ols is highly dependent on their degree of polymerization [6]. Polymeric flavan-3-ols (also known as proanthocyanidins) have a large molecular size and increased hydrophilicity, thereby having difficulty in penetrating the lipid bilayer of the small intestine [6]. Proanthocyanidins therefore primarily undergo microbial biotransformation in the colon [7]. Among the proanthocyanidin subclasses, procyanidin has been widely studied, as it is the most common type found in foods including cocoa, chocolate, pine bark extract and grape seed extract [7,8]. Procyanidin is exclusively composed of (epi)catechins and is further classified into A and B types, depending on the presence of an additional ether bond [7]. Although A- and B-type procyanidins have a different chemical structure, metabolomics analyses of both types have identified common metabolites such as (epi)catechins, 3-(3′,4′-dihydroxyphenyl)-propionic acid (DHPA), and 3,4-dihydroxyphenyl-acetic acid (DHAA) [9,10]. 5-(3′,4′-dihydroxyphenyl)-γ-valerolactone (DHPV) has been recognized as the major microbial metabolite of procyanidins and (epi)catechins [10,11]. Previous studies have shown that DHPV and its conjugated forms are detected in plasma and urine, at low µM concentrations [12,13,14,15] and ~40 µmol during 24 h [13] respectively, after the intake of various proanthocyanidin-rich foods. In this study, we sought to investigate DHPV in comparison with procyanidin derivatives including A- and B-type procyanidin dimers and other major microbial metabolites for its preventive effect on CVDs using a monocyte-endothelial cell adhesion model. Major procyanidin derivatives were selected based on previous studies [6] (Figure 1).

## 2. Results

### 2.1. 5-(3′,4′-dihydroxyphenyl)-γ-Valerolactone (DHPV) Exerts Superior Preventive Effects against Tumor Necrosis Factor (TNF)-α Stimulated THP-1 Monocyte-Endothelial Adhesion Compared to other Procyanidin Derivatives

**Figure 2:**
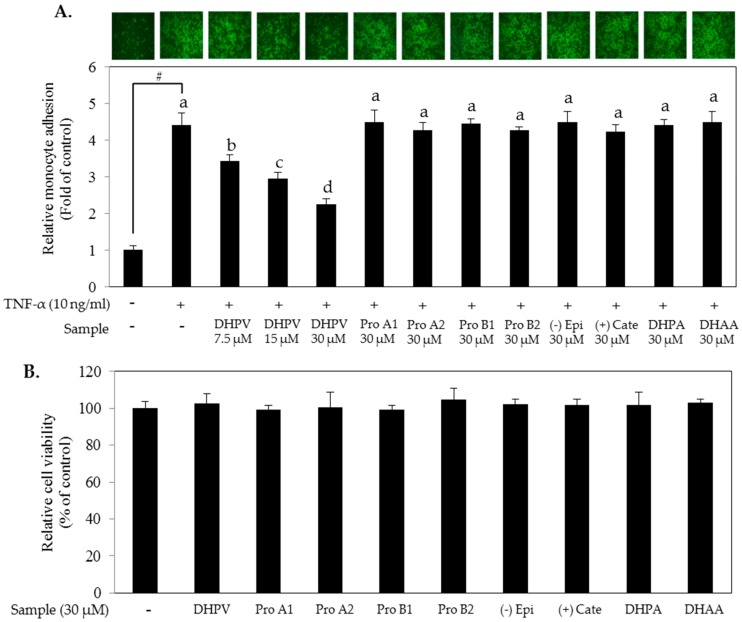
Comparison of monocyte-endothelial adhesion inhibitory effects between DHPV and other procyanidins and their microbial metabolites. (**A**) Following pretreatment of procyanidin derivatives at the indicated concentrations for 1 h, human umbilical vein endothelial cells (HUVECs) were exposed to tumor necrosis factor (TNF)-α for a further 5 h. Calcein AM-labeled THP-1 human monocytic cells were then added to the HUVEC medium and for 1 h to allow for adherence. The number of adhered THP-1 cells was determined with a fluorescence microplate reader at excitation and emission wavelengths of 485 and 535 nm, respectively. Images were captured with a fluorescence microscope; (**B**) Varying concentrations of DHPV and 30 μM of various procyanidins and their microbial metabolites were tested for cytotoxicity. Data are expressed as means ± SD (*n* = 4). # *p* < 0.05 versus control. Different letters (a, b, c and d) denote significant differences (*p* < 0.05); values bearing the same letters are not significantly different from each other. Pro A1, procyanidin A1; Pro A2, procyanidin A2; Pro B1, procyanidin B1; Pro B2, procyanidin B2; (−) Epi, (−) epicatechin; (+) Cate, (+) catechin; DHPA, 3-(3′,4′-dihydroxyphenyl)-propionic acid; DHAA, 3,4-dihydroxyphenyl-acetic acid.

Major procyanidin derivatives were selected based on previous studies (Figure 1). To investigate the preventive effect of the compounds against atherosclerosis, we pretreated human umbilical vein endothelial cells (HUVECs) with 30 μM of procyanidin dimers (Pro A1, Pro A2, Pro B1 and Pro B2), monomers ((−) epicatechin and (+)-catechin) or their microbial metabolites including DHPV, DHPA and DHAA for 1 h prior to the tumor necrosis factor (TNF)-α stimulation. Among the selected procyanidin derivatives, DHPV significantly prevented the adhesion of THP-1 monocytes to the TNF-α-stimulated HUVECs in a concentration-dependent manner (Figure 2A). Concentrations of the procyanidin derivatives up to 30 μM did not compromise cell viability (Figure 2B).

### 2.2. DHPV Attenuates TNF-α-Stimulated Upregulation of VCAM-1 Protein and mRNA Expression

**Figure 3:**
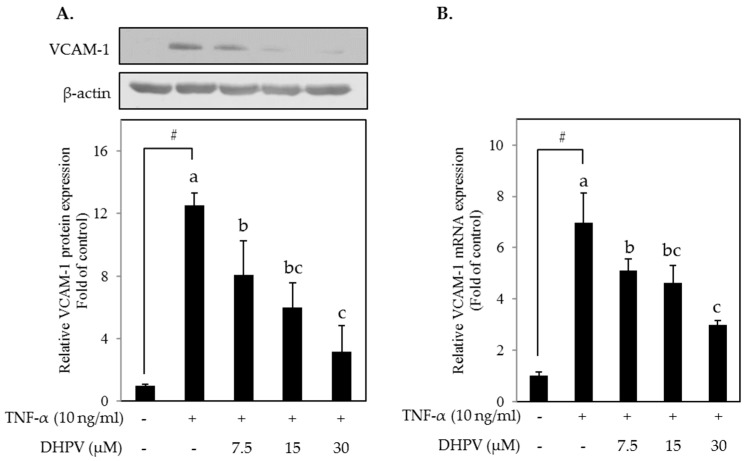
Effect of DHPV on vascular cell adhesion molecule (VCAM-1) expression in TNF-α-stimulated HUVECs. (**A**) Preventive effect of DHPV on TNF-α-stimulated VCAM-1 protein expression as assessed by Western blot. The image of the band is the representative image and data are expressed as means ± SD (*n* = 3); (**B**) VCAM-1 mRNA expression was measured by RT-qPCR. Data are expressed as means ± SD (*n* = 4). # *p* < 0.05 versus control. Different letters denote significant differences (*p* < 0.05); values bearing the same letters are not significantly different from each other.

We sought to evaluate whether DHPV exerts preventive effects on TNF-α-stimulated THP-1 monocyte-endothelial adhesion through the suppression of vascular cell adhesion molecule (VCAM)-1. Western blot analysis and RT-qPCR showed that DHPV inhibited VCAM-1 expression at both the mRNA and protein levels in TNF-α-stimulated HUVECs in a concentration-dependent manner (Figure 3).

### 2.3. DHPV Attenuates TNF-α-Stimulated Upregulation of MCP-1 Protein Secretion and mRNA Expression

**Figure 4:**
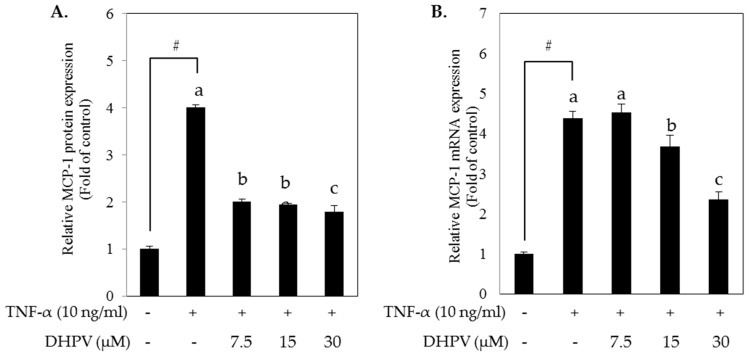
Effect of DHPV on MCP-1 expression in TNF-α-stimulated HUVECs. (**A**) Preventive effect of DHPV on TNF-α-stimulated MCP-1 protein secretion as assessed by ELISA; (**B**) MCP-1 mRNA expression was analyzed by RT-qPCR. Data are expressed as means ± SD (*n* = 4). # *p* < 0.05 versus control. Different letters denote significant differences (*p* < 0.05); values bearing the same letters are not significantly different from each other.

After the monocyte adhesion to vascular endothelium, monocytes migrate into vascular intima by the stimulation of monocyte chemoattractant protein (MCP)-1 protein. To investigate whether DHPV treatment can prevent this migration, we performed enzyme-linked immunosorbent assay (ELISA) to quantify MCP-1 protein in the culture media supernatants from the TNF-α-stimulated HUVECs. DHPV pretreatment attenuated TNF-α-stimulated MCP-1 secretion as well as mRNA expression in HUVECs (Figure 4).

### 2.4. DHPV Suppresses TNF-α-Stimulated Activation of NF-κB Promoter in HUVECs

**Figure 5:**
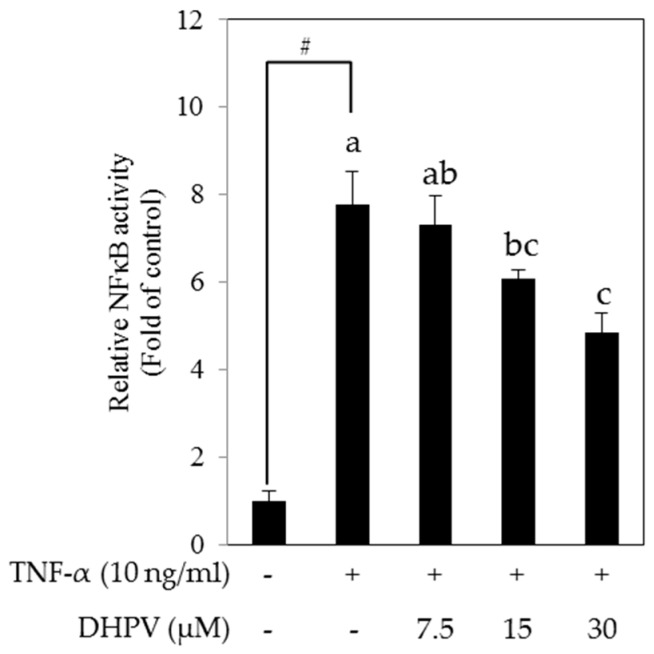
Effect of DHPV on TNF-α-induced NF-κB transcriptional activity. Following pretreatment with DHPV and TNF-α-stimulation, NF-κB transcriptional activity was measured in HUVECs transfected with NF-κB luciferase reporter plasmids. Data are expressed as means ± SD (*n* = 4). # *p* < 0.05 versus control. Different letters denote significant differences (*p* < 0.05); values bearing the same letters are not significantly different from each other.

To determine whether the preventive effect of DHPV on TNF-α-stimulated expression of VCAM-1 and MCP-1 is associated with the inhibition of NF-κB, we analyzed NF-κB transactivation in HUVECs stably transfected with NF-κB luciferase reporter plasmids. Pretreatment with DHPV significantly suppressed TNF-α-stimulated transcriptional activation of NF-κB in a concentration-dependent manner (Figure 5).

### 2.5. DHPV Suppresses TNF-α-Stimulated Phosphorylation of Proteins Involved in NF-κB Signaling in HUVECs

**Figure 6:**
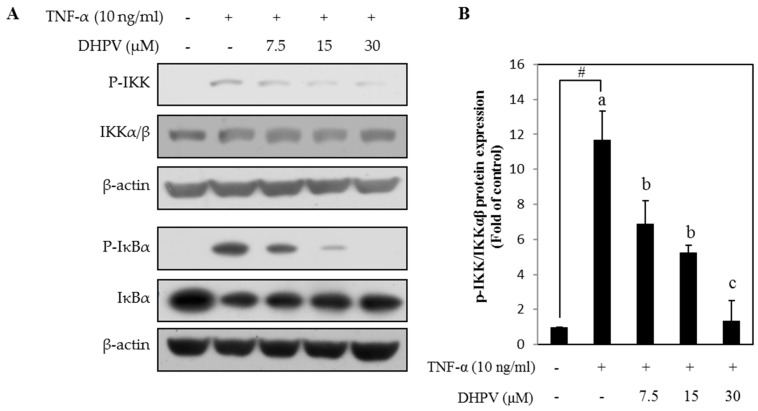
Effect of DHPV on TNF-α-stimulated NF-κB signaling. Expression of phosphorylated and unphosphorylated proteins comprising the NF-κB signaling pathway as determined by Western blot. (**A**) Representative image; (**B**) Quantification data of phosphorylated I kappa-B kinase (IKK), the upstream signaling protein of IκBα. Data are expressed as means ± SD (*n* = 3). # *p* < 0.05 versus control. Different letters denote significant differences (*p* < 0.05); values bearing the same letters are not significantly different from each other.

To further elucidate the mechanism how DHPV modulates NF-κB activity, we examined the preventive effect of DHPV on TNF-α-induced phosphorylation of I kappa-B kinase (IKK) and IκBα, the two key regulators of NF-κB activation signaling. We observed that DHPV downregulated TNF-α-stimulated phosphorylation of IKK in a concentration-dependent manner as well as IκBα, a downstream effector of IKK (Figure 6). DHPV also prevented degradation of IκBα following TNF-α-stimulation.

## 3. Discussion

Several metabolomics studies have shed light on the potential metabolic pathways responsible for the microbial catabolism of procyanidins (Figure 1) [6]. Gut microflora can cleave the strong interflavan bonds within procyanidins to produce (epi)catechin-monomer building blocks [16] with a slow reaction rate [10,17]. It appears that the A- and C-rings in (epi)catechins and procyanidins are primarily targeted by microbiota [7,10]. Two human intestinal bacterial species, *Eggerthella lenta* rK3 and *Eubacterium* (E.) sp. Strain SDG-2, were found to cleave C-rings in (epi)catechins [18,19]. In addition, *Flavonifractor plautii* aK2 further converts the C-ring-cleaved intermediate into DHPV via A-ring breakdown and lactone formation [19]. DHPV may also be directly derived from breakdown of the A-ring in procyanidins [10]. DHPV is further degraded into DHPA and DHAA via β- and α-oxidation, respectively [7]. These metabolites have been found in human biological fluids, and procyanidin dimers such as A2 [20], B1 [21] and B2 [22], (epi)catechins [23], DHPV [12,13,14,15] and DHAA [24,25] have been identified in human blood plasma after intake of procyanidin-rich foods. In contrast to DHPA and DHAA, which can be derived from the microbial catabolism of other flavonoid subclasses [26], DHPV has been exclusively detected in human biological fluids after consumption of (epi)catechin-rich green tea [15], proanthocynidin-rich cocoa [12,27], and pycnogenol [28]. Despite the importance of DHPV in procyanidin catabolism, little research has been focused on the properties of DHPV, such as its anti-inflammatory activity. DHPV exhibits stronger inhibitory activity than its precursor catechin on lipopolysaccharide-induced nitric oxide production in RAW 264.7 macrophages and the key enzymes of inflammatory and degenerative disorders, such as the matrix metalloproteinases (MMPs) [8,29]. In addition, DHPV exhibits more potent radical scavenging and antioxidative activities than catechin and vitamin C [29]. The present study marks the first report of the potential cardioprotective effects of DHPV.

Atherosclerosis is a major cause of CVDs and is thought to be highly preventable, particularly through adherence to a healthy diet [30]. In the early stages of atherosclerosis, vascular endothelial cells under inflammatory conditions begin to attract leukocytes such as monocytes [31,32,33]. It has been well established that VCAM-1 and MCP-1 in the vascular endothelial cells are the key drivers of monocyte-endothelial adhesion [34]. VCAM-1 integrin is reported to be the critical mediator that strongly binds monocytes [33]. After integrin-monocyte binding, inflammatory conditions continue to activate the chemokine MCP-1, which further attracts monocytes into the vascular intima [33]. Proinflammatory cytokines such as TNF-α stimulate a phosphorylation cascade within the IκB/NF-κB pathway, and the translocation of NF-κB into the nucleus activates transcription of numerous genes, including pro-inflammatory cytokines, adhesion molecules and chemokines [32,33,34]. Akt and mitogen-activated protein kinase signaling is another possible pathway which can regulate the expression of VCAM-1 in endothelial cells [35]. As curcumin inhibited TNF-α-stimulated VCAM-1 expression through this pathway, it is possible that a molecular target of DHPV is associated with this pathway.

So far, DHPV and its conjugated forms are detected in plasma at low µM concentrations after the intake of various proanthocyanidin-rich foods in previous studies [12,13,14,15]. In our experiments, 7.5 µM of DHPV significantly inhibited most of the TNF-α-stimulated pro-inflammatory responses except the mRNA expression of MCP-1 (Figure 4B) and the NF-κB transcriptional activity (Figure 5). The cause of this error is possibly due to two reasons, the first being the different time points for harvesting protein, mRNA, and luciferase samples. The media supernatant samples for ELISA assay in Figure 4A were harvested 5 h after the TNF-α stimulation, the mRNA samples for RT-qPCR in Figure 4B were after 4 h, and the samples for luciferase assay in Figure 5 was after 10 h. Second, the different sensitivities of the detection methods. For example, comparing the effect of DHPV at 30 µM in Figure 5 and Figure 6, the NF-κB promoter activity in Figure 5 was not reduced to the control level as the NF-κB signaling pathway in Figure 6. The data of luciferase assay indirectly reflect the activation of NF-κB promoter during 10 h by accumulated luciferase in cells, while the data of Western blot show phosphorylation levels of IKK and IκBα at 15 min after the TNF-α stimulation. Additionally, since HUVECs are primary cultured cells, cells with lower passage number might have higher sensitivity to DHPV. However, considering all these variations of experiments, it is still clear that 15 µM of DHPV significantly inhibited TNF-α-stimulated pro-inflammatory responses, which lead us to assume that the minimum concentration of DHPV to show preventive effect in our model will be between 7.5 and 15 µM.

It has been previously reported that frequent consumption of procyanidin-rich cocoa improves vascular endothelial function and reduces levels of soluble adhesion molecules in the plasma of individuals who are at a high-risk of CVDs [36,37]. Several other in vivo and in vitro studies have shown that procyanidin-rich extracts reduce levels of VCAM-1 and MCP-1 under inflammatory conditions [38,39,40]. Other studies focusing on (epi)catechin metabolites have reported that both epicatechin phase II metabolites [41] and rat plasma after intake of (+)catechin [42] inhibit monocyte-endothelial adhesion. In the case of DHPV, a single study has shown that the plasma of human volunteers containing DHPV after intake of pycnogenol inhibits NF-κB activation and MMP-9 secretion in activated human monocytes [43]. The present study marks the first report showing that DHPV has the most potent preventive effects on TNF-α-stimulated THP-1 monocyte-endothelial adhesion among the procyanidin derivatives, downregulates two prominent biomarkers of atherosclerosis (VCAM-1 and MCP-1), activates NF-κB transcription, and phosphorylates IKK and IκBα. Further kinase targets of DHPV need to be identified in order to elucidate its full effects on atherosclerosis prevention.

## 4. Materials and Methods

### 4.1. Chemicals and Reagents

5-(3′,4′-dihydroxyphenyl)-γ-valerolactone (DHPV), purity 95%, was purchased from Chemieliva Pharmaceutical (Chondquing, China). Referring to Ottaviani et al.’s study [13], we briefly decided the concentration range of DHPV between the amount of DHPV conjugates detected in plasma to urine assuming that urine amount per day is 1 L. Procyanidin A1 (Pro A1) was purchased from Phytolab (Vestenbergsgreuth, Germany). Procyanidin A2 (Pro A2) and Procyanidin B1 (Pro B1) were purchased from Extrasynthese (Genay, France). Procyanidin B2 (Pro B2) was purchased from Funakoshi (Tokyo, Japan). (+)catechin, (−)epicatechin, 3-(3,4-dihydroxyphenyl)propionic acid (DHPA), 2-(3,4-dihydroxyphenyl)acetic acid (DHAA), fetal bovine serum (FBS), medium 199 (M199), hydrocortisone, 2-mercaptoethanol, puromycin and calcein acetoxymethyl (AM) solution were purchased from Sigma-Aldrich (St. Louis, MO, USA). RPMI 1640 medium was purchased from Welgene (Daegu, Korea). Recombinant human epidermal growth factor (hEGF), basic fibroblast growth factor (bFGF) and l-glutamine were purchased from Gibco (Grand Island, NY, USA). Recombinant human tumor necrosis factor-α (TNF-α) was purchased from PeproTech Korea (Seoul, Korea). Penicillin (10,000 units/mL)-streptomycin (10,000 µg/mL) (P/S) was purchased from Corning (Corning, NY, USA). Antibodies against vascular cell adhesion protein-1 (VCAM-1), β-actin and total I kappa-B kinase (IKK)α/β were purchased from Santa Cruz Biotechnology (Santa Cruz, CA, USA). Phosphorylated IKKα/β and IκBα and total IκBα were purchased from Cell Signaling Biotechnology (Danvers, MA, USA). 3-(4,5-dimethylthiazol-2-yl)-2,5-diphenyltetrazolium bromide tetrazolium salt (MTT) solution was purchased from USB Corporation (Cleveland, OH, USA).

### 4.2. Cell Culture

Human umbilical vein endothelial cells (HUVECs) were purchased from Lonza (Walkersville, MD, USA) and grown in M199 supplemented with 25 mM 4-(2-hydroxyethyl)-1-piperazineethanesulfonic acid containing 10% (*v*/*v*) FBS (Gibco, Grand Island, NY, USA), 2 mM l-glutamine, 1 ng/mL hydrocortisone, 1% (*v*/*v*) P/S and the two growth factors hEGF (1 ng/mL) and bFGF (2 ng/mL). Stably-transfected HUVECs harboring NF-κB luciferase reporter plasmids were cultured with M199 containing 1 μg/mL puromycin. HUVECs between passages 7 and 12 were used. THP-1 cells, which consist of monocyte-like cells derived from a leukemia line, were purchased from the Korean Cell Line Bank and cultured in RPMI 1640 media supplemented with 10% (*v*/*v*) FBS (Sigma-Aldrich, St. Louis, MO, USA), 50 µM 2-mercaptoethanol and 1% (*v*/*v*) P/S. Subculturing occurred when the density reached between 2 × 10^5^ and 1 × 10^6^/mL.

### 4.3. Cell Viability Assay

In 96-well plates, confluent HUVECs were starved with serum-free M199 medium containing 2 mM l-glutamine for 4 h. The starved HUVECs were treated with various concentrations of procyanidin derivatives dissolved in dimethylsulfoxide (DMSO). After 22 h incubation, MTT solution was added to the medium at 0.5 mg/mL. At 24 h, 200 µL DMSO was added, and the absorbance at 570 nm was measured after 30 min incubation.

### 4.4. THP-1 Monocyte Adhesion Assay

In 96-well plates, confluent HUVECs were starved for 4 h and treated with procyanidin derivatives in M199 supplemented with 2 mM l-glutamine and 10% (*v*/*v*) FBS for 1 h and then stimulated with 10 ng/ml TNF-α for 5 h. THP-1 cells were stained with calcein AM and added to the HUVECs at 5 × 10^5^ cells/well in M199. After 1 h incubation, non-adhered THP-1 cells were washed off with phosphate-buffered saline (PBS), and the adhered cells were assessed for florescence using an Infinite 200 PRO (Tecan group Ltd., Männedorf, Switzerland) at excitation and emission wavelengths of 485 and 538 nm, respectively.

### 4.5. Western Blot Assay

Confluent HUVECs in 6-well plates or 6 cm dishes were starved for 4 h and treated with DHPV at up to 30 μM for 1 h. Pre-treated cells were stimulated with 10 ng/mL TNF-α for 15 min (NF-κB signaling) or 5 h (VCAM-1) and harvested with radioimmunoprecipitation assaybuffer after cold PBS washing. Quantified protein lysate samples were separated in 10% sodium dodecyl sulfate-polyacrylamide gels and transferred onto polyvinylidene difluoride membranes. The membranes were blocked and incubated with specific primary antibodies at 4 °C overnight. Horseradish peroxidase-conjugated secondary antibodies were added for 1 h, and the protein bands were then visualized using an enhanced chemiluminescence detection kit (GE Healthcare, London, UK).

### 4.6. Enzyme-Linked Immunosorbent Assay for MCP-1

Following 4 h of cell starvation, 1 h of DHPV pretreatment, and 5 h of TNF-α-stimulation, protein levels of monocyte chemoattractant protein-1 (MCP-1) in the HUVEC culture supernatant were measured using Human MCP-1/CCL2 ELISA MAX Deluxe Sets (BioLegend, San Diego, CA, USA). Briefly, 100 μL of standard cytokines or diluted culture supernatants were added to each well of an MCP-1 antibody-coated 96-well plate and incubated for 2 h at room temperature. Each well was incubated with the detection antibody for 1 h followed by Avidin-HRP solution together with the substrate solution for 30 and 20 min, respectively. Appropriate washing was performed between each addition of solution. The optical density of each well was determined using a microplate reader at 450 and 570 nm. A standard curve for the cytokine was generated, and linear regression analysis was performed.

### 4.7. Real-Time Quantitative PCR

Following 4 h of cell starvation, 1 h of DHPV pretreatment, and 4 h of TNF-α-stimulation, total RNA was extracted from HUVECs using Trizol and RNA iso Plus (Takara Bio Inc., Shiga, Japan), and quantified using a NanoDrop ND-2000 spectrophotometer (Thermo Fisher Scientific, Waltham, MA, USA). cDNAs were synthesized from a total of 1 μg/μL RNA using a PrimeScriptTM 1st strand cDNA Synthesis Kit (Takara Bio Inc., Shiga, Japan). Glyceraldehyde 3-phosphate dehydrogenase (GAPDH) was selected as an internal standard. The cDNAs were probed using the following primers (Bioneer, Daejeon, Korea): human VCAM-1 forward (5′-CCCTCCCAGGCACACACA-3′); human VCAM-1 reverse (5′-GATCACGACCATCTTCCCAGG-3′); human MCP-1 forward (5′-TCGCCTCCAGCA TGAAAGTC-3′); human MCP-1 reverse (5′-GGCATTGATTGCATCTGGCT-3′); human GAPDH forward (5′-CAGGGCTGCTTTTAACTCTGGTAAA-3′); human GAPDH reverse (5′-GGGTGGAATCATATTGGAACATGTAA-3′). For quantitative real-time PCR, iQTM SYBR Green^®^ Supermix and a CFX ConnectTM Real-time PCR Detection System (Bio-Rad Laboratories, Hercules, CA, USA) were used.

### 4.8. Luciferase Assay for NF-κB Transactivation

The lentiviral expression vector pGF1-NF-κB-EF1-Puro (System Biosciences, Palo Alto, CA, USA) was transfected into HEK293T cells with the packaging vectors pMD2.G and psPAX2 (Addgene, Cambridge, MA, USA) using JetPEI DNA transfection reagent (Polyplus-transfection, New York, NY, USA). HUVECs were transfected using filtered transfection medium harvested from the HEK293T cell culture and 10 μg/mL polybrene (EMD Millipore, Billerica, MA, USA). After selection with 1 μg/mL puromycin (InvivoGen, San Diego, CA, USA) for 24 h, stably transfected HUVECs were seeded at 1 × 10^4^ cells/well in 96-well plates. After 4 h of starvation, the cells were pretreated with DHPV at up to 30 μM for 1 h before stimulation with 10 ng/mL TNF-α. After 10 h stimulation, the cells were disrupted with lysis buffer (0.1 M pH 7.8 PBS, 1% Triton X-100, 1 mM dithiothreitol and 2 mM ethylenediaminetetraacetic acid, and luciferase activity was measured using a Luminoskan Ascent (Thermo Electron, Helsinki, Finland).

### 4.9. Statistical Analysis

Statistical analyses were performed using SPSS (Statistical Analysis System Institute, 2010). Significant differences between the means were determined using Tukey’s Honest Significant Difference test at *p* < 0.05.

## 5. Conclusions 

Metabolomics studies have supported the notion that DHPV is a biologically important microbial metabolite exclusively derived from flavan-3-ols. Several DHPV-producing gut microbiota have been identified as well. Our findings suggest that DHPV may be a primary contributor toward the preventive effects of procyanidins on CVDs that have been observed in clinical studies. Further studies on the reduced risk of CVDs from chronic procyanidin intake and the presence of DHPV-producing microbiota, correlating with bioavailable DHPV levels in plasma are warranted.

## Abbreviations

CVDs	Cardiovascular diseases
DHPV	5-(3′,4′-dihydroxyphenyl)-γ-valerolactone
DHPA	3-(3′,4′-dihydroxyphenyl)-propionic acid
DHAA	3,4-dihydroxyphenyl-acetic acid
Pro	Procyanidin
HUVECs	Human umbilical vein endothelial cells
VCAM-1	Vascular cell adhesion protein-1
TNF-α	Tumor necrosis factor-α
MCP-1	Monocyte chemoattractant protein-1
IKK	Iκ-B kinase
